# Antioxidant and Immunomodulatory Activity of the Alkaloidal Fraction of *Cissampelos pareira* Linn.

**DOI:** 10.3797/scipharm.0904-16

**Published:** 2009-12-06

**Authors:** Anand Bafna, Shrihari Mishra

**Affiliations:** 1 Dr. D. Y. Patil Institute of Pharmaceutical Sciences and Research, Pimpri, Pune-411018, India; 2 The M. S. University of Baroda, Vadodara-390002, Gujarat, India

**Keywords:** Alkaloidal fraction, Humoral antibody titre, Superoxide, Lipid peroxidation, *Cissampelos pareira* Linn

## Abstract

The alkaloidal fraction (AFCP) of roots of *Cissampelos pareira* Linn. was screened for *in-vitro* antioxidant activity and immunomodulatory activity in mice. The HPTLC finger print profile was also established for the identification of AFCP which was found to contain 0.176 % of berberine. AFCP possess strong antioxidant activity which was revealed by its ability to scavenge the stable free radical DPPH, superoxide ion and to inhibit lipid peroxidation in rat liver homogenate induced by iron/ADP/Ascorbate complex. AFCP was found to have significant immunosuppressive activity at lower doses (25 and 50 mg/kg) while no activity was observed at higher doses (75 and 100 mg/kg). Humoral antibody titre was significantly (p<0.01) lowered by AFCP at the doses of 25 and 50 mg/kg. Delayed type hypersensitivity response was also significantly (p<0.01) suppressed by the AFCP at the dose of 75 mg/kg. Thus the present study revealed the immunosuppressive and antioxidant activities of the alkaloidal fraction of *C. pareira* roots.

## Introduction

Immunomodulation using medicinal plants, especially ‘rasayana’ drugs, can provide an alternative to conventional chemotherapy for a variety of diseases, especially when the host defense mechanism has to be activated under the conditions of impaired immune response or when a selective immunosuppression is desired in situations like autoimmune disorders. This concept of using rasayanas for health, gained little more credibility, when it was realized that herbal antioxidants concurrently exhibit significant immunomodulatory activities, like Shilajit and Chyavanprash Awaleha [1]. Further, Indian medicinal plants are a rich source of substances which are claimed to induce innate immunity, the non-specific immunomodulation of essentially granulocytes, macrophages, natural killer cells and complement functions [2].

About 34 plants are identified as rasayanas in Indian Ayurvedic system of medicine having various pharmacological properties such as immunostimulant, tonic, neurostimulant, antiageing, antibacterial, antiviral, antirheumatic, anticancer, adaptogenic, antistress, antioxidant etc. Many plants with potential immunomodulatory and antioxidant activities are reported, some of these have already been undertaken for evaluation of their activities in animals, and also to some extent in humans. Some glaring examples with promising activity are *Asparagus racemosus, Azadirachta indica, Curcuma longa, Ocimum sanctum, Panax ginseng, Picrorrhiza kurroa, Tinospora cordifolia, Withania somnifera* etc. A lot more are still to be explored and offer scope for further investigation [3].

*Cissampelos pareira* Linn. (Menispermaceae) is a climbing shrub distributed throughout warm parts of Asia, East Africa, and America. The roots are used as a diuretic and febrifuge, as a remedy for heart trouble, dysentery and soares [4]. Furthermore, the roots are also used to prevent a threatened miscarriage and the herb is used to stop uterine hemorrhage [5]. A novel tropoloisoquinoline alkaloid named pareirubrine A was reported for antileukemic activity [6]. Pradhan et al carried out pharmacological and clinical studies on hayatin methiodide from *C. pareira* for its muscle relaxant properties [7]. Basu et al reported curare like activity of hyatinin methochloride from *C. pareira* [8]. Cissamperine and other four bisbenzylisoquinoline alkaloids isolated from *C. pareira* were found to show significant and reproducible inhibitory activity against human carcinoma of the nasopharynx cell culture (KB) [9].

The roots of this plant are mainly incorporated into many traditional Ayurvedic formulation prescribed for diseases like rheumatism, ulcers, fevers etc. Our earlier work reported the immunomodulatory activity of methanol extract of *C. pareira* [10]. Alkaloids from roots of this plant were mainly screened for various pharmacological activities and in order to correlate immunomodulatory activity with alkaloids the present work was aimed at studying effect of alkaloidal fraction of *C. pareira* on immune system as well as its ability to scavenge free radicals.

## Results and Discussion

Present investigation was undertaken to mainly evaluate the antioxidant and immunomodulatory activities of one of the rasayana drug *C. pareira* using some reported methods.

### In-vitro antioxidant activity

Free radical scavenging activity of the AFCP was evaluated using different models. Inhibition of lipid peroxidation in rat liver homogenate was also evaluated. Table 1 shows the DPPH scavenging effect of AFCP. AFCP showed a concentration dependent antiradical activity by inhibiting DPPH radical with an IC_50_ value of 63.44 μg/mL. This activity was comparable to the standard curcumin, which showed an IC_50_ at 52.71 μg/mL.

AFCP was also found to scavenge the superoxide radical generated in riboflavin-NBT-light system *in-vitro* and IC_50_ value was found to be 31.99 μg/mL. It was less potent than the standard ascorbic acid which showed an IC_50_ of 23.52 μg/mL (Table 2).

In the present study AFCP showed moderate inhibition of lipid peroxidation induced by Iron/ADP/Ascorbate complex in rat liver homogenate. The IC_50_ value was found to be 61.85 μg/mL and 30.05 μg/mL for AFCP and standard ascorbic acid respectively (Table 3). AFCP showed a dose dependent inhibition of lipid peroxidation.

The participation of reactive oxygen species in etiology and physiopathology of human diseases, such as neurodegenerative disorders, inflammation, viral infection, autoimmune pathologies and digestive system disorders such as gastrointestinal inflammation and gastric ulcers is already evident. To understand the role of these reactive oxygen species in several disorders and potential antioxidant protective effect of natural compounds on affected tissues are topics of high current interest. Initially it is necessary to investigate *in-vitro* antioxidant properties of any natural product or drug to consider it as an antioxidant substance, followed by evaluation of its antioxidant function in biological systems [11]. AFCP showed strong *in vitro* antioxidant activity in tested models viz., scavenging of DPPH and superoxide radicals and inhibition of lipid peroxidation induced by Iron/ADP/Ascorbate complex in rat liver homogenate.

Tetrandrine [12] and berberine [13] were previously isolated from *C. pareira* roots. They were also reported for their antioxidant activity in various models [14, 15]. AFCP was found to contain 0.176 % of berberine and activity of AFCP may be due to presence of these two with other alkaloids. This antioxidant activity of the AFCP may be responsible for various biological activities of the drug, including proposed immunosuppressive activity.

### Immunomodulatory activity

Recently, many isoquinoline alkaloids are under intensive study for their antimicrobial, anti-tumor, neuropharmacological and immunosuppressive action. Some of them are already in clinical use and it is of interest to investigate their possible effect on the immune system [16].

The effect of AFCP was tested on humoral and cell-mediated immunity by measuring haemagglutination antibody titre and DTH response respectively. The effect was tested at four different dose levels ranging from 25 to 100 mg/kg.

Results obtained during present investigation showed significant (p<0.01) reduction in antibody production in response to SRBCs at doses 25 and 50 mg/kg. With further increase in dose AFCP had no suppressive effect on antibody production and values obtained were more or less equal to control animals (Table 4).

Suppression of production of antibodies by AFCP at lower doses was comparable to positive control cyclophosphamide. The positive control group showed significant (p<0.01) reduction of antibody production.

AFCP when administered orally showed a linear dose dependent decrease in DTH response up to 75 mg/kg, however statistically significant decrease could be obtained at 75 mg/kg dose (p<0.01). At 100 mg/kg of the AFCP the DTH response was slightly increased (Table 4). The positive control, cyclophosphamide group showed increased DTH response when compared to control group.

The present results indicated that AFCP was an effective modulator of both T and B cell mediated immune response. The antibody production to T-dependent antigen SRBCs requires the co-operation of T and B-lymphocytes and macrophages [17]. Results obtained during present investigation showed significant suppressive effect on antibody production in response to SRBCs at lower doses of the AFCP, whereas no further decrease was noted with increase in doses i.e. at higher doses no effect on HA titre was noted.

DTH is antigen specific and causes erythema and induction at the site of antigen infection in immunized animals. T-cells are required to initiate the reaction. The histology of DTH can be different for different species, but the general characteristics are an influx of immune cells at the site of injection, macrophages and basophils in mice and induction becomes apparent within 24–72 hours [18].

Treatment with cyclophosphamide showed reduced humoral antibody titre and enhancement of DTH. Cyclophosphamide has a particularly intense effect on short lived lymphocytes known to include a great proportion of B-cells [19].

*C. pareira* mainly contains bisbenzoylisoquinoline alkaloids. In more recent research, the bisbenzylisoquinoline alkaloids have been found to be anti-inflammatory constituents of *C. pareira.* In clinical experiments, the alkaloids suppressed the production of nitric oxide, a critical mediator in inflammation, which explain some aspects of the anti-inflammatory mechanisms present in the alkaloids of *C. pareira* [20]. Results obtained were in accordance with this. AFCP suppressed DTH response in a dose dependent manner.

In the past few years high performance thin layer chromatography has emerged as a potential tool for rapid and useful phytochemical evaluation of herbal drugs [21]. Establishment of HPTLC fingerprint profile for the AFCP would help to identify this fraction of *C. pareira.*

Thus from the present study, it can be concluded that AFCP has a strong *in vitro* antioxidant activity and also possess immunosuppressive activity.

## Material and Methods

### Plant material

Roots of *C. pareira* Linn. were collected from outfield of Baroda city, Gujarat, India. They were authenticated in the Botany Department of M. S. University, Baroda and voucher specimen was submitted there.

### Preparation of alkaloidal fraction (AFCP)

Air dried roots were subjected to extraction with methanol by maceration for 72 hours. Yield obtained was 7.75 % (w/w). This methanol extract was utilized for preparation of alkaloidal fraction. Methanol extract was acidified with 150 mL of 5% hydrochloric acid and left overnight at room temperature. The insoluble non-alkaloid material was removed by filtration. The filtrate was subjected to ethyl ether extractions to eliminate the rest of the non-alkaloid substances. The residues obtained after evaporation of ether extracts were added to non-alkaloid fraction. The acidic solution thus purified was then alkalized with 25% ammonium hydroxide and extracted with chloroform (6x 200 mL). The evaporation of the combined chloroform extracts gave alkaloidal fraction (AFCP), which was further used for screening antioxidant activity and immunomodulatory activities.

### Animals

Swiss albino mice of either sex, weighing 20–25 g, housed in standard conditions of temperature, humidity and light were used. They were fed with standard rodent diet and water ad libitum. Albino rats of either sex, weighing 150–180 g, for preparation of liver homogenate.

### Drugs

Weighed quantity, 50 mg/10 mL of AFCP was suspended in 1% sodium carboxy methylcellulose to prepare suitable dosage form. The control animals were given an equivalent volume of sodium carboxy methylcellulose vehicle.

Antigen: Fresh blood was collected from sheep which were sacrificed in local slaughterhouse.

Sheep red blood cells (SRBC) were washed three times in normal saline and adjusted to a concentration of 20% for immunization and 1% for challenge.

### HPTLC finger print profile of AF and quantitative analysis of berberine

HPTLC finger print profile was established for AFCP of *C. pareira using* CAMAG-HPTLC. A stock solution (1mg/mL) of AFCP was prepared in methanol. Standard berberine solution (0.1 mg/mL) was also prepared in methanol. Calibration curve was obtained for 10, 20, 30 and 40 ng concentrations of berberine. The solvent system used was n-butanol: glacial acetic acid: water (6:2:2). The plates were scanned using TLC Scanner 3 (CAMAG) at 366 nm (fluorescence/reflectance mode)

**Fig. 1.**
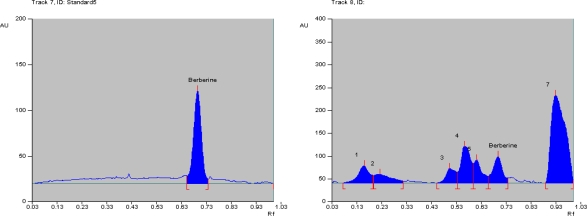
Chromatogram of standard berberine and AFCP.

HPTLC finger print profile was established for AFCP of *C. pareira using* CAMAG-HPTLC. A stock solution (1 mg/mL) of AFCP was prepared in methanol. Standard berberine solution (0.1 mg/mL) was prepared in methanol. Calibration curve was obtained for 10, 20, 30 and 40 ng concentration of berberine. The solvent system used was *n*-butanol: glacial acetic acid: water (6:2:2). The plates were scanned using TLC Scanner 3 (CAMAG) at 366 nm (fluorescence/reflectance mode) and after spraying Dragendorff’s reagent at 520 nm. Berberine was detected in AF at Rf value 0.70. Chromatogram and peak areas of resolved bands were recorded and photographs were taken (Fig. 1). Concentration of berberine in sample found was 0.1763 % and after spraying Dragendorff’s reagent at 520 nm. Berberine was detected in AFCP at Rf value of 0.70. Chromatogram and peak areas of resolved bands were recorded and photographs were taken (Fig. 1). The concentration of berberine in sample was found to be 0.1763 % w/w.

## Methods

### In-vitro antioxidant activity

#### Assay for antiradical activity with DPPH

Antiradical activity was measured by a decrease in absorbance at 516 nm of a methanolic solution of colored 1,1-diphenyl-2-picrylhydrazine brought about by sample. A stock solution of DPPH was prepared by dissolving 4.4 mg in 3.3 mL methanol. Test medium includes 150 μL of DPPH solution along with different concentration (20, 40, 60, 80 and 100 μg/mL) of samples in 3 mL methanol. Blank was performed in the same way with no sample added. Decrease in absorbance, in presence of sample was noted after 15 minutes. An IC_50_ was calculated as the concentration which brought about a 50% reduction in absorbance compared to blank [22].

#### Assay for superoxide radical scavenging activity

The assay was based on capacity of the sample to inhibit blue formazon formation by scavenging the superoxide radicals generated in riboflavin-light-nitro blue tetrazolium (NBT) system. The reaction medium contains 2.5 mL of phosphate buffer (pH 7.6), 100 μL riboflavin (20 μg), 200 μL EDTA (12mM), 100μL NBT (0.1 mg) and different concentrations (10, 20, 30, 40 and 50 μg/mL) of sample contained in 100 μL of methanol. The reaction was started by illuminating the reaction mixture for 5 minutes. The absorbance was measured at 590 nm. Blank was performed in the same way with 100 μL of methanol instead of test substance. An IC_50_ was calculated as the concentration which brought about a 50% reduction in absorbance compared to blank [23].

#### Measurement of effect on lipid peroxidation on rat liver homogenate

Rat liver homogenate was prepared by homogenizing the tissue in chilled Tris buffer (10 mM, pH 7.4) at a concentration of 10% w/v. Lipid peroxidation was induced in the liver homogenate by Iron-ADP complex in the presence of ascorbic acid. The incubation medium constituted of 0.5 mL of the liver homogenate (10% w/v), 100 μM FeCl_3_, 1.7 μM ADP, 500 μM of ascorbate and different concentrations of samples in 2 mL of total incubation medium. The medium was incubated for 20 min. at 37°C. Extent of lipid peroxidation was determined by estimation of malondialdehyde (MDA) content. Results were expressed in terms of decrease in MDA formation by the sample extract. Ascorbic acid was used as the positive control [24].

### Immunomodulatory activity

#### Humoral antibody (HA) response

Animals were divided into 6 groups of six animals each. Animals in treatment the groups were given the AFCP (25-100 mg/kg, p.o.) in 1.0 % sodium carboxy methyl cellulose daily for 7 days. Animals in positive control group received 50 mg/kg of cyclophosphamide (CP) for days 4 to 6. The animals were immunized by injecting 0.1 mL of 20% of fresh sheep red blood cells suspension intraperitonially on day 0. Blood samples were collected in micro centrifuge tubes from individual animal by retro-orbital plexus on day 7. Blood samples were centrifuged to obtain serum. Antibody levels were determined by haemagglutination technique. The reciprocal of the highest dilution of the test serum which caused agglutination was taken as the antibody titre [25].

#### Delayed type hypersensitivity (DTH) response

Animals were divided into 6 groups of six animals each. Animals in treatment groups were given the AFCP (25–100 mg/kg, p.o.) in 1.0 % sodium carboxy methyl cellulose daily for 7 days. Animals in positive control group received cyclophosphamide (CP) 50 mg/kg for day 4 to 6. The animals were immunized by injecting 0.1 mL of 20% of fresh sheep red blood cells suspension intraperitonially on 0 day. On day 7, the thickness of the right hind foot pad was measured using digital vernier caliper. The mice were then challenged by injection of 20 μL of 1% SRBCs in right hind foot pad. Foot thickness was again measured 24 hrs after this challenge. The difference between the pre and post challenge foot thickness expressed in mm was taken as a measure of DTH [25].

## Figures and Tables

**Fig. 2.**
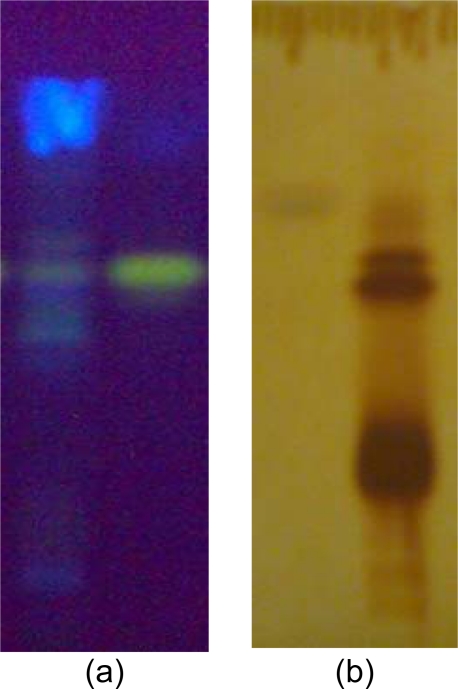
Photograph of berberine and AFCP at 366 nm (a) and with dragendorff’s reagent (b).

**Tab. 1.** Antiradical activity of AFCP observed with DPPH.

**Samples**	**Concentration (μg/mL)**	**% inhibition**	**IC_50_ (μg/mL)**
Alkaloidal fraction	20	24.35 ± 2.37	63.44

	40	36.34 ± 1.23	

	60	53.98 ± 3.54	

	80	59.70 ± 1.89	

	100	66.39 ± 2.05	

Curcumin			52.71

Values are mean ± S.E.M. of three replicate analyses.

**Tab. 2.** Superoxide anion scavenging activity of AFCP observed with a riboflavin-light-NBT system.

**Samples**	**Concentration (μg/mL)**	**% inhibition**	**IC_50_ (μg/mL)**
Alkaloidal fraction	10	14.54 ± 1.59	31.99

	20	35.62 ± 2.29	

	30	48.69 ± 1.99	

	40	62.35 ± 2.54	

	50	74.28 ± 1.16	

Ascorbic acid			23.52

Values are mean ± S.E.M. of three replicate analyses.

**Tab. 3.** Inhibition of lipid peroxidation induced by iron/ADP/ascorbate system in rat liver homogenate by AFCP.

**Samples**	**Concentration (μg/mL)**	**% inhibition**	**IC_50_ (μg/mL)**
Alkaloidal fraction	25	25.38 ± 2.55	61.85

	50	45.67 ± 1.96	

	75	60.24 ± 2.89	

	100	75.02 ± 2.22	

	125	80.29 ± 3.57	

Ascorbic acid			30.05

Values are mean ± S.E.M. of three replicate analyses.

**Tab. 4.** Effect of AFCP on humoral antibody titre and DTH response.

**Treatment**	**Dose (mg/kg)**	**HA Titre (Mean ± S.E.M.)**	**DTH (Mean ± S.E.M.)**
Control	–	149.33 ± 35.70	0.38 ± 0.08

CP	50	10.00 ± 2.68**	1.07 ± 0.10**

Alkaloidal fraction	25	5.00 ± 1.00**	0.31 ± 0.01^NS^

	50	8.00 ± 1.79**	0.23 ± 0.02^NS^

	75	64.00 ± 14.31^NS^	0.15 ± 0.04**

	100	133.33 ± 40.83^NS^	0.27 ± 0.02^NS^

F value		7.30	4.01

P value		<0.0005	<0.012

Values are expressed as mean ± SEM.

Control group was compared with Cyclophosphamide (CP) group and AFCP treated groups.

* p<0.05;

** p<0.01; NS = Non Significant
